# Nail Unit Specimens

**DOI:** 10.3390/dermatopathology13030043

**Published:** 2026-09-09

**Authors:** Curtis T. Thompson

**Affiliations:** 1 CTA Pathology, Beaverton, OR 97008, USA; curtis@ctapathology.com; 2 Department of Dermatology, University of Miami Miller School of Medicine, Miami, FL 33136, USA; 3 Departments of Dermatology and Pathology, Oregon Health and Science University, Portland, OR 97239, USA

**Keywords:** biopsy, microscopy, histology, tissue, sectioning, orientation, cassette, formalin

## Abstract

Histologic submission and processing of nail unit specimens require special attention, primarily because of the contrast between the hard plate and the delicate matrix/bed tissue. Adherence of the plate to the glass slide is particularly challenging, and a protocol is included to address this challenge. Loss of the often small, fragile matrix/bed tissue can lead to absence of histopathologic features needed to make a definitive diagnosis. Of primary importance is the submission of matrix/bed specimens, which are often small and fragmented on a paper template of a nail, which is then placed securely in a tissue cassette between sponges, thereby preserving tissue orientation and allowing for flat fixation for a minimum of 8 h. Immunostains and other special stains also differ somewhat from their use elsewhere on the skin, the details of which are presented.

## Simple Summary

Histologic submission and processing of nail unit specimens require special attention. Adherence of the plate to the glass slide is particularly challenging. Loss of the often small, fragile matrix/bed tissue makes diagnoses difficult. Submission of matrix/bed specimens on a paper template is important. Immunostains and other special stains also differ somewhat from their use elsewhere on the skin. Protocols are presented to aid in nail unit specimen processing.

## 1. Introduction

Much of the trepidation that histologists and histopathologists have about nail specimens is centered around the challenge of preparing adequate histologic slides, particularly when a pigmented lesion is being sampled. A central challenge of processing is centered around the hard plate which is therefore difficult to section and adhere to a glass slide. In contrast, matrix/bed specimens are small and delicate and can be easily damaged or lost. In addition, orientation is challenging, particularly when the biopsy is from an abnormal or diseased nail unit. A few simple techniques employed by the nail surgeon and by the histology laboratory can greatly simplify the process and produce high-quality histologic sections [1].

**Figure 1:**
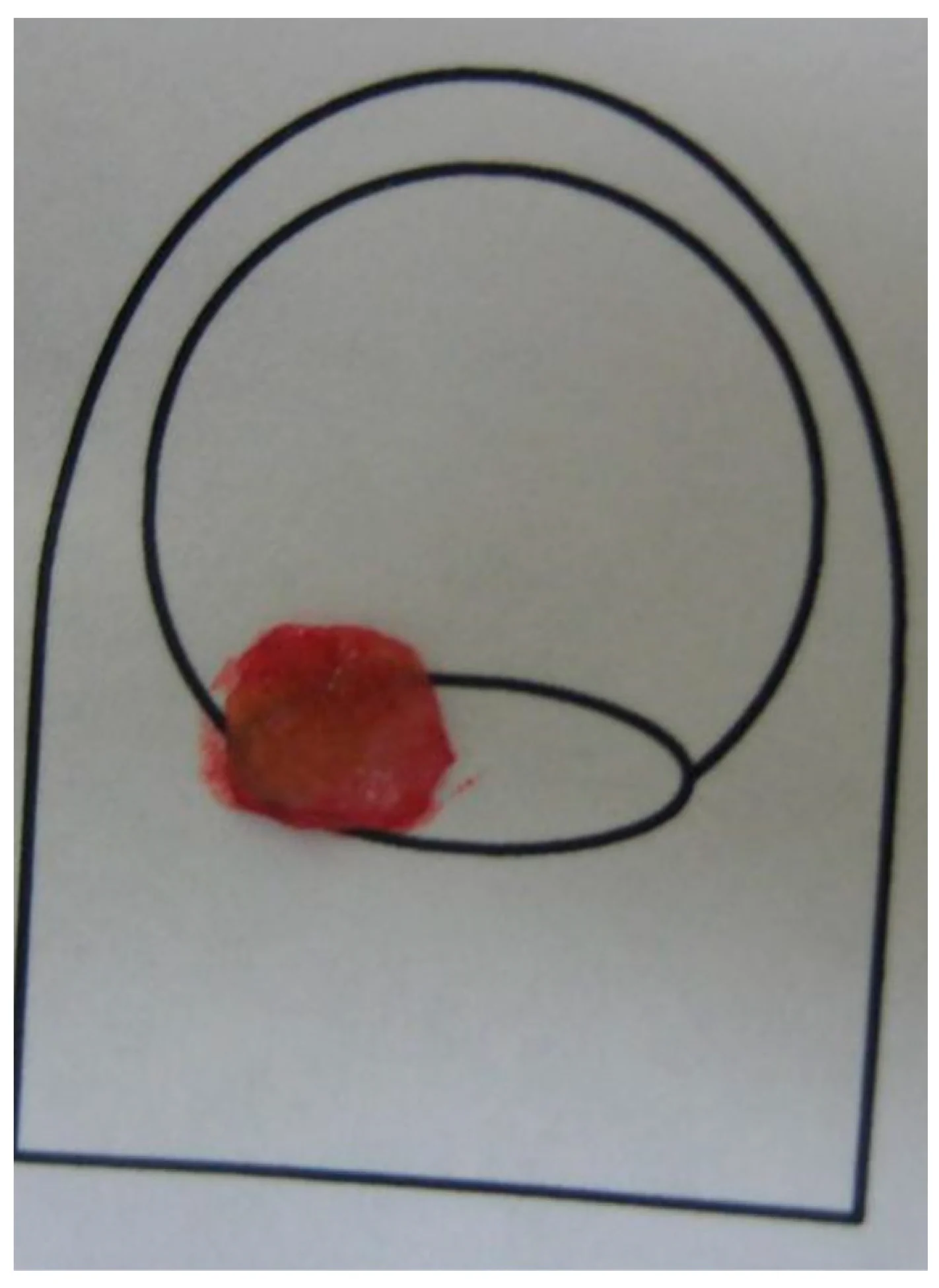
A matrix/bed specimen after removal being placed on a paper template in the same orientation as in vivo.

This guide aims to present simple protocols for submission and processing of nail unit specimens. The protocols have been developed and refined in the author’s laboratory over 20 years. Therefore, individual parameters may require adjustment based on local laboratory conditions. For submission of a matrix/bed specimen, the technique described below incorporates concepts from protocols used in other tissue pathology, particularly ophthalmologic pathology, wherein small, delicate specimens are submitted to the laboratory on a paper template, in a manner which preserves anatomic orientation [2]. Margin assessment is easier with this technique. By submitting the specimen on a paper template of a nail, the delicate matrix/bed tissue fixes flat while maintaining orientation for proximal/distal and medial/lateral anatomy (Figure 1).

The following concepts are important in submitting nail specimens to pathology:

- Using a protocol for plate processing for better adherence of the plate to the glass slide;
- Submitting an oriented, intact specimen to the laboratory for precise grossing, sectioning and embedding;
- Providing a clinical history with a differential which guides the pathologist and the laboratory in the preparation and analysis of each specimen.

## 2. Nail Plate Processing

One of the greatest challenges in nail specimen analysis is the processing of the hard plate so that it remains adhered to the glass slide. A wide variety of techniques exist, with most of these protocols developed in individual histologic laboratories, which are rarely assessed in a controlled manner [3]. As such, a plethora of these homebrew techniques exist [4]. The use of a strong acid or base to pretreat and soften the tissue (i.e., hair removal cream, fabric conditioner, 30% KOH) can aid in sectioning; however, it can mute the antigenicity needed for immunostaining [5]. Using charged slides and pre-coating them with gelatin are important steps in plate adherence to the glass slide.

**Figure 2:**
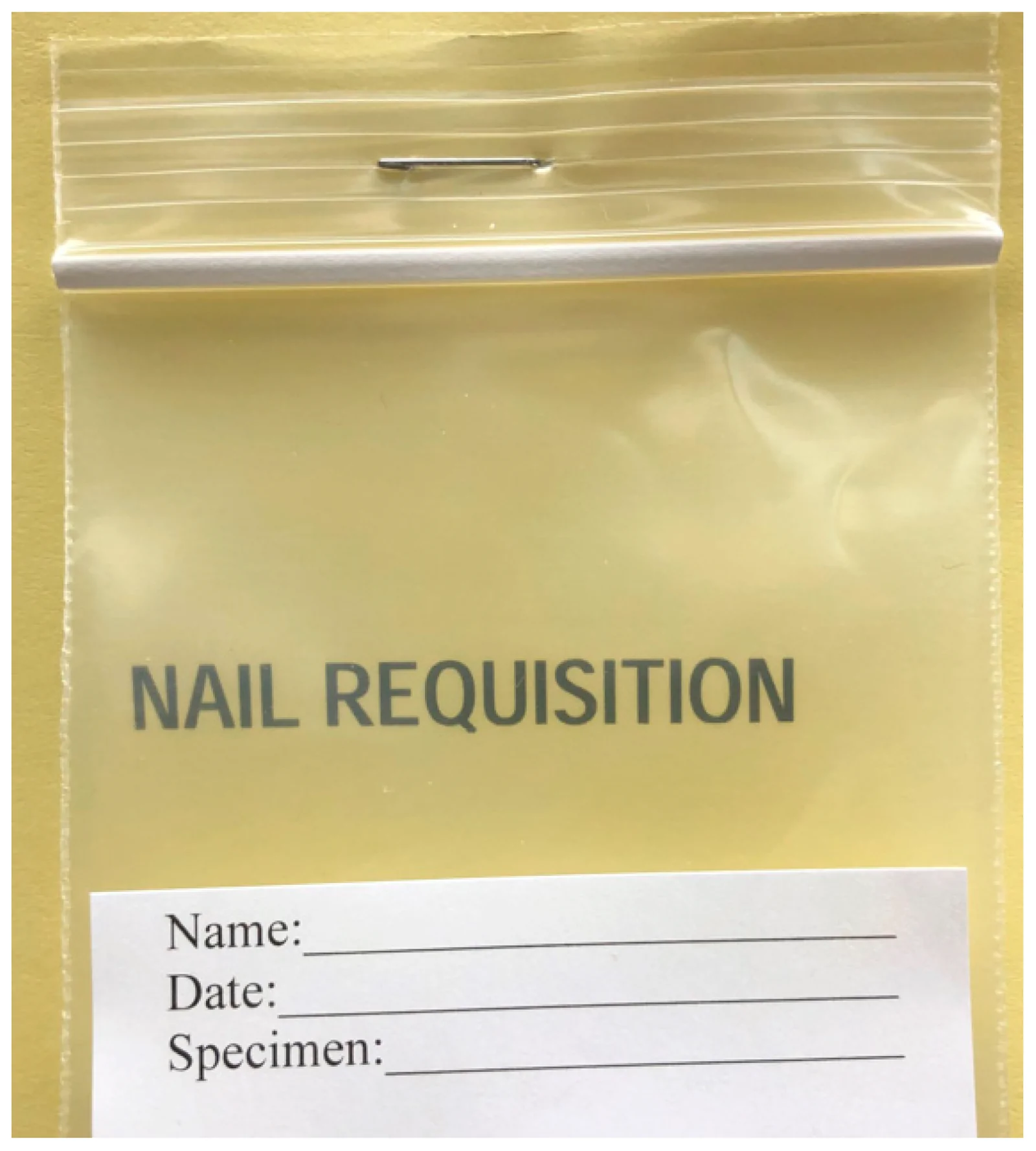
A small, zipper storage bag attached to a requisition for deposition of nail plate fragments, which should especially include subungual debris.

When the plate alone is submitted, usually for fungal or pigment (blood, melanin, etc.) identification, the following items should be considered:

- Submit the plate dry in a small zipper storage bag, and include subungual debris (Figure 2).
- For fungal identification, PCR, if available, may offer higher sensitivity, as demonstrated in some studies, than traditional PAS and other traditional staining techniques [6].
- If PCR is not available, only larger nail plate fragments, which will survive histologic processing, should be submitted for H&E and PAS staining.
- PAS and GMS (Grocott’s methenamine silver) stains show similar sensitivity [7].
- If the subungual debris is not submitted for PCR examination and the PAS stain is negative, a Cytospin^TM^ (Epredia, 4481 Campus Dr, Kalamazoo, MI 49008) PAS staining technique may be considered to increase sensitivity (technique described below) [8].

A small, zipper storage bag attached to the specimen requisition is particularly useful. Subungual debris is lost when the specimen is placed in an empty bottle or one containing formalin. Larger plate fragments can undergo usual overnight histologic processing, and the subungual debris can be reserved for possible centrifuge collection of the subungual debris, culture or PCR. The following protocol works well for plate processing to ensure adherence to the glass slide.

## 3. Protocol for Nail Plate Fragments Histologic Processing and PAS Staining

1. Place gelatin stock solution in water bath (final concentration 0.01–0.03%).
2. Cut a 4 μm ribbon, and float it in a gelatin water bath.
3. If the embedded plate fragments are too hard to cut a ribbon, the face of the tissue block may be soaked in decalcification solution to soften the fragments. The soaking time is variable, depending upon the hardness of the plate.
4. Using a positively charged slide, pick up the desired sections for the PAS and H&E slides.
5. Place the slides in a 65 C oven for 45 min (longer for ‘difficult’ specimens).
6. Deparaffinize the slides using xylene or xylene substitutes, and hydrate through an alcohol series (or place the PAS slides on a programmed deparaffinization run and H&E slides on an H&E program on an auto stainer).
7. For the PAS slides, gently rinse the slides in running tap water.
8. Rinse the slides in distilled water.
9. Do not digest the slide, as the tissue will fall off the slide.
10. Place the slides in 1% periodic acid for 10 min.
11. Gently rinse the slides quickly in distilled water.
12. Place the slides in Schiff’s solution for 10 min.
13. Gently rinse the slides in warm–hot tap water for 5 min.
14. Place the slides in light green stain as needed to reach desired background intensity (approximately 30 s).
15. Dehydrate the slides through an alcohol series.
16. Clear the slides through three changes of xylene or a xylene substitute.
17. Coverslip using a permanent mounting medium.

**Figure 3:**
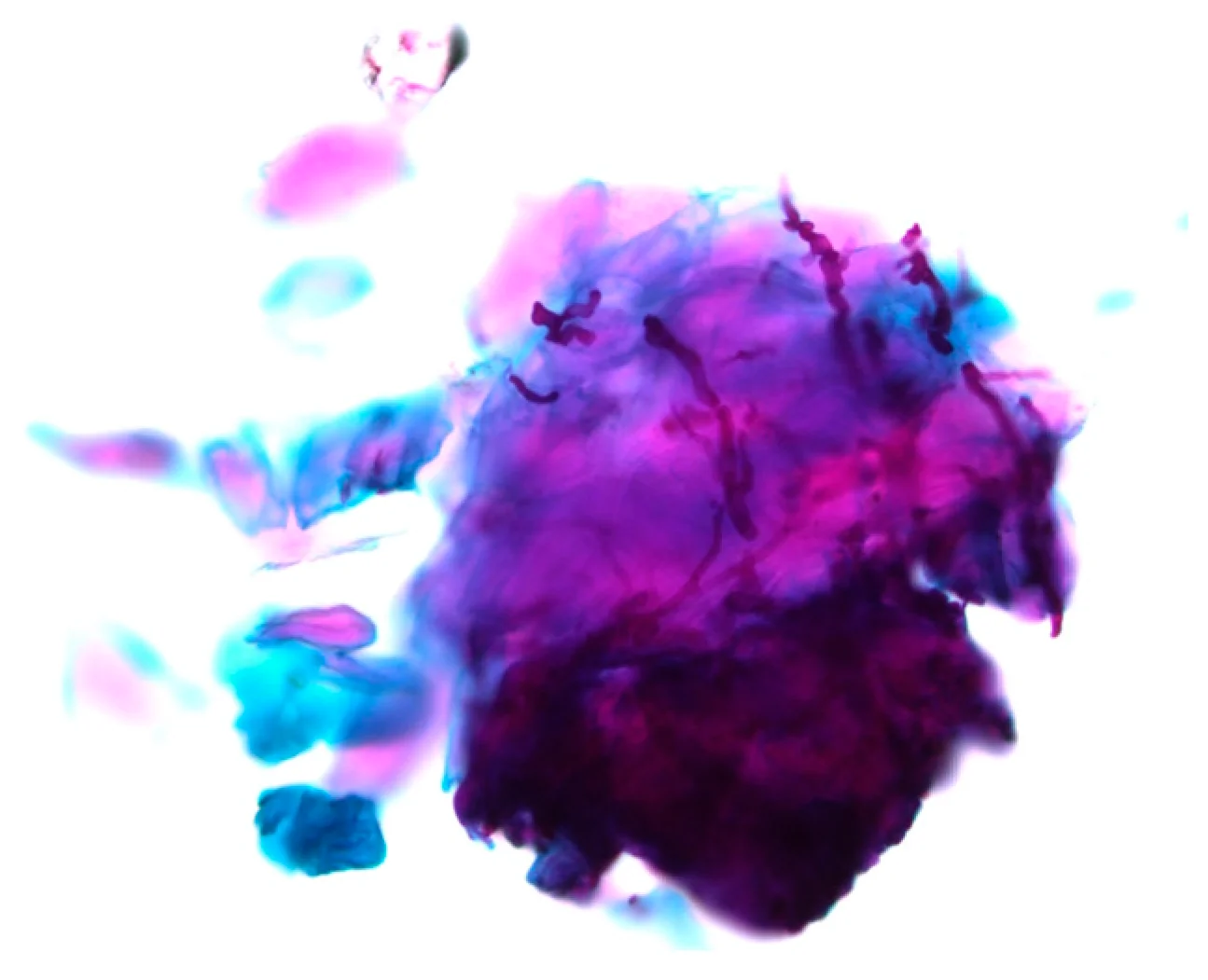
Hyphal forms identified using a cytospin preparation of subungual debris (PAS stain, 400×). The protocol for the technique is presented as follows.

If the initial PAS stain is negative on the larger nail fragments, the subungual debris can be processed and analyzed. It is possible to isolate subungual debris from a sample in formalin; however, this requires centrifugation of all of the formalin, a process that is burdensome and not necessary if the specimen is submitted dry. A protocol for detecting subungual debris is quite easy in the laboratory as long as the proper centrifuge system is used (Cytospin^TM^, (Epredia, 4481 Campus Dr, Kalamazoo, MI 49008, USA). It is important to note that this places all of the subungual debris from the sample onto one glass slide on which a PAS stain can be performed. Of note, the microscopic analysis of the PAS slide can be burdensome, as it is not uncommon to identify a single focus of hyphal forms with this method (Figure 3).

## 4. Cytospin^TM^ Protocol for PAS Staining of Subungual Debris

**Materials Needed (Other than usual histology supplies):**

Calibrated pipette (1–5 mL)

Disposable pipette

Cytospin^TM^ centrifuge (Epredia, 4481 Campus Dr, Kalamazoo, MI 49008, USA) 

Cytospin^TM^ funnel/holder (Epredia, 4481 Campus Dr, Kalamazoo, MI 49008, USA)

1. Using the calibrated pipette, dispense 1 mL of 10% neutral buffered formalin into the debris container, and agitate to suspend the nail debris.
2. Using the disposable pipette, dispense the debris/formalin solution into a labeled culture test tube.
3. Place the capped culture test tube into the centrifuge, and spin for 5 min.
4. Using the disposable pipette, draw off some of the supernatant (about ½ of it), taking care not to disturb the debris pellet at the bottom of the test tube.
5. Replace the cap, and centrifuge for another 5 min.
6. Assemble the Cytospin^TM^ funnel with the labeled positively charged slide and holder.
7. Using a disposable pipette, dispense the remaining supernatant and the nail debris pellet from the test tube into the funnel.
8. Centrifuge 11 min to completion.
9. Remove the funnel/holder with the slide, and air-dry the slide for 5 min.
10. Place the slide in 95% alcohol for 1 min in a Coplin jar.
11. Air-dry the slide for 10 min.
12. Stain with PAS using the nail PAS protocol (above).

## 5. Fungal/Yeast Speciation and Assessment for a Mold Using Culture or PCR

Though traditional tissue culture identifies species of fungus, it is insensitive and should not be used as a primary screen for a fungal infection, since it is much less sensitive than PCR or a PAS stain [6,9]. If culture must be used, it is important to notify the microbiology laboratory that a mold is a consideration so that the culture may be performed using a cycloheximide-free medium. Unfortunately, there is high variability among microbiology laboratories in their ability to culture fungus.

## 6. Identification of Blood in the Nail Plate

**Figure 4:**
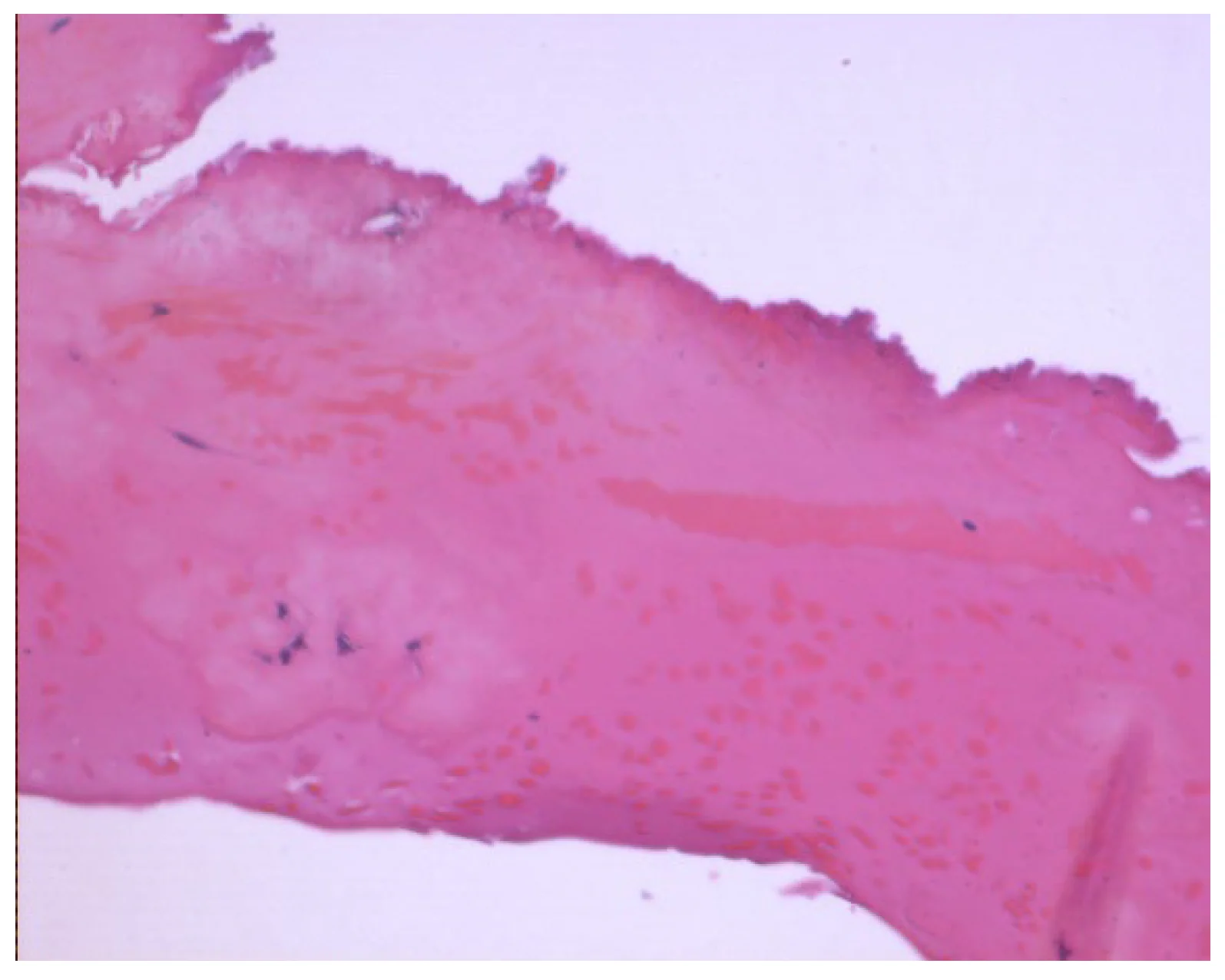
Blood within a nail plate, identifiable on H&E sections. A Perl’s iron stain is unnecessary (H&E, 100×).

One of the most common reasons that the plate alone is sampled is when the patient presents with a pigmented lesion, and the clinician wants to identify the source of the pigment, particularly to rule out melanoma. Trauma is the primary cause of nail plate hemorrhage, but the patient often does not report a history of overt trauma. Of particular note is that the blood in the plate does degrade, and the iron remains a part of the heme group. Thus, a Perl’s iron stain does not stain blood within the plate. Fortunately, the blood is identifiable on H&E sections (Figure 4) [10].

## 7. Nail Matrix/Bed Specimens

After a general skin biopsy or excision, skin specimens are routinely placed free-floating in a container of formalin. If orientation is needed, a suture may be placed on the tissue, or the tissue may be marked with a cut or ink. With nail unit specimens, however, especially specimens of the delicate matrix/bed, placing the specimen free-floating in formalin leads to the specimen becoming fixed in an awkward shape with loss of orientation or fragmentation during transport. One particular challenge in matrix/bed sampling arises when the clinician observes various, irregular areas of pigmentation upon reflecting the nail plate. In cases like this, the shave specimen of the matrix/bed must often be removed in multiple pieces.

The techniques and protocols described below help overcome these limitations. By maintaining the integrity of the nail unit specimen, the process of grossing and sectioning is greatly streamlined, and the pathologist has a much better chance at arriving at a definitive diagnosis.

A laboratory which processes nail specimens regularly is ideal, but medical systems are now quite large and varied, so submission of the specimen in a way that allows for precise processing in different laboratories is ideal. Communication between the clinician and the laboratory is important, with both needing to pay close attention to all of the fragments of tissue removed. This is particularly important when sampling a pigmented lesion, since histologic identification of the pigmentation may be focal and subtle.

Because small matrix/bed specimens are often depleted with initial sectioning, it is important for the laboratory to perform initial H&E level sections with intervening unstained sections. Section thickness is the same as other small skin specimens. Refacing of the tissue block for later sections is not ideal. Also, ensuring that the clinician has provided a clear clinical history with a differential is important, so that grossing and initial sections can attempt to show specific features of the different nail lesions. Without a differential, such as “Rule-out onychopapilloma,” a pathologist may end up incorrectly rendering a diagnosis of “verruca/wart,” since benign epithelial neoplasms of the nail unit often have hyperkeratosis and hypergranulosis, features of a verruca.

## 8. A Simple Technique for Submission of Nail Matrix/Bed Samples

**Figure 5:**
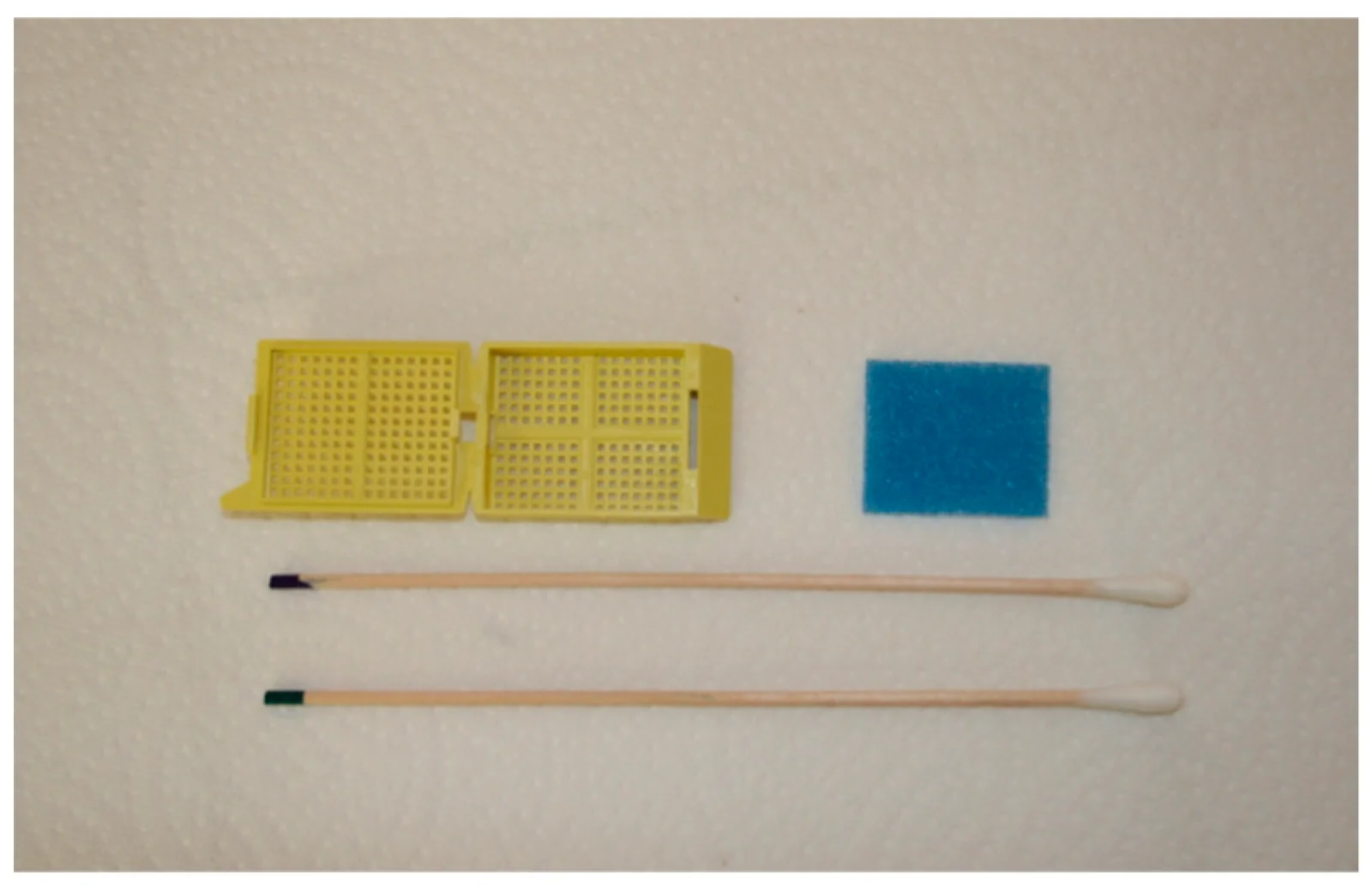
Tools from the histopathology laboratory which are necessary for submitting an oriented matrix/bed biopsy.

Using a nail template and simple equipment from the histopathology laboratory (Figure 5), a clinician can submit a specimen in a protected and oriented manner.

**Figure 6:**
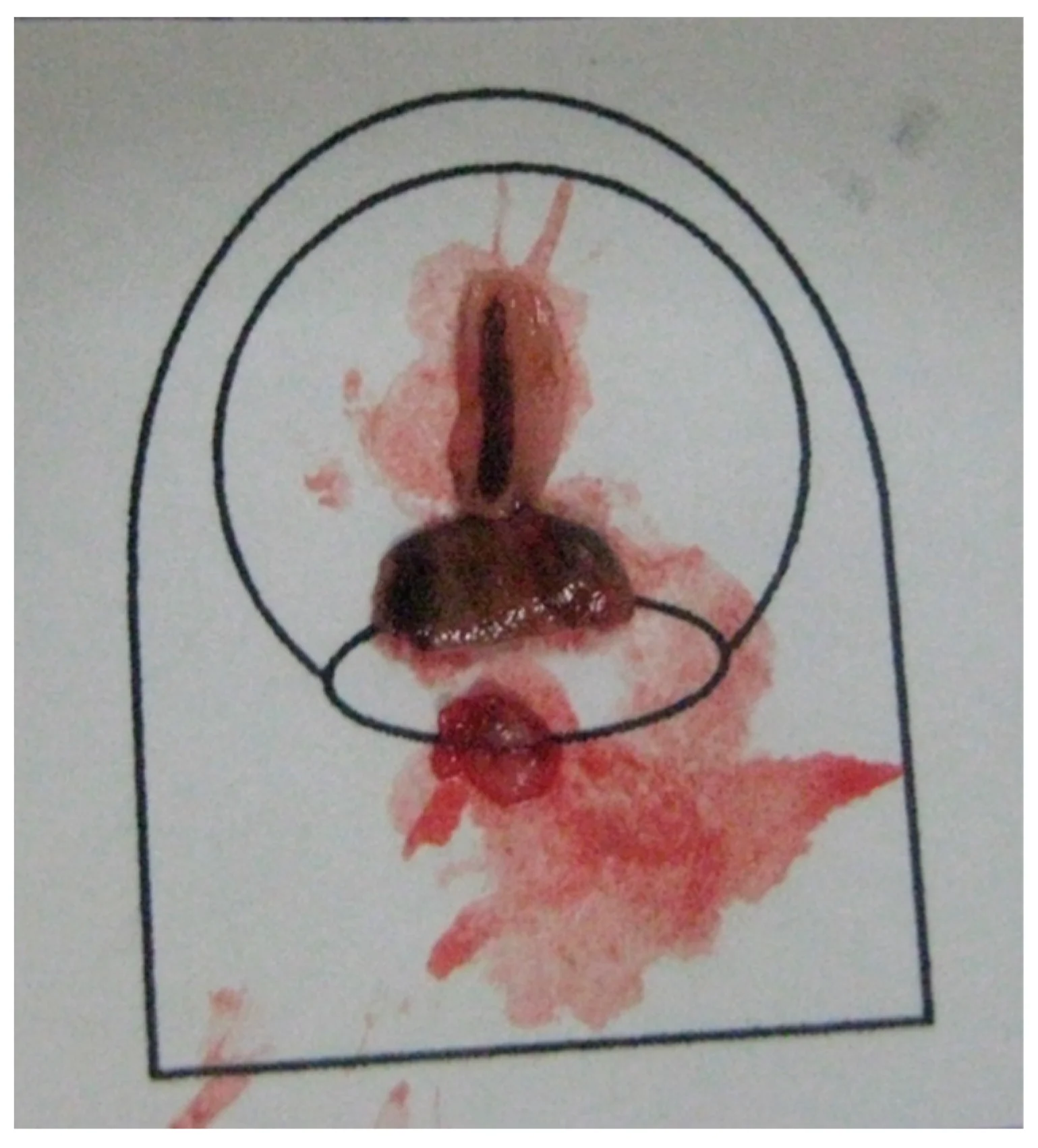
A pigmented lesion of the matrix/bed, sampled in multiple pieces and placed in an oriented manner on the paper template.

Borrowing from ophthalmologic specimen processing [2], the specimen is oriented by placing it on a paper template print-out of a nail, with the specimen in the same location as its in vivo location (Figure 6).

Any type of paper may be utilized for the template, and the template may even be drawn by hand with a pencil. A template print-out may be found at https://ctapathology.com/wp-content/uploads/2025/11/Revised-Nail-Diagram-Pre-Processing.pdf (accessed on 3 September 2026). The template paper should be wetted before the specimen is placed on it to prevent histologic drying artifact. The template with the specimen should fit easily within the tissue cassette so the cassette does not pop open. The specimen should be fixed in formalin for a minimum of 8 h before it is sent for processing.

Orientation may be maintained by inking one or more edges of the specimen and the corresponding template paper. Precise inking is best achieved using the wooden end of a cotton swab rather than the cotton end. Because many lesions are pigmented, avoiding black ink is important to prevent confusion of ink with melanin. Thus, green or blue ink is best.

**Figure 7:**
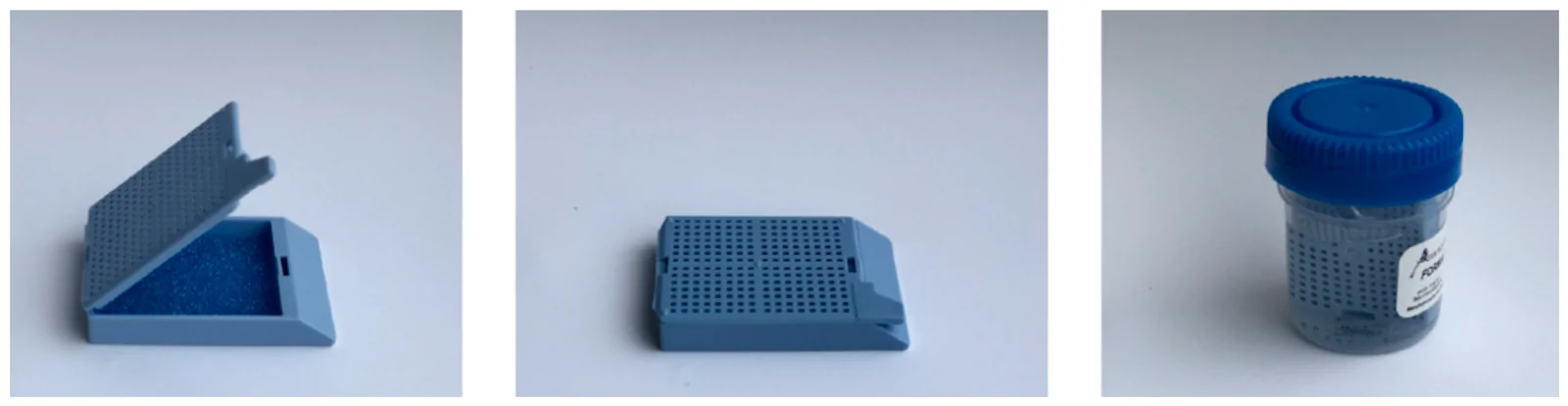
A cassette with the template, specimen and sponge is closed and placed in a larger specimen bottle.

The specimen on the template print-out is then placed in a tissue cassette between two sponges and placed in formalin (Figure 7). A supply of tissue cassettes and the sponges can be given to the clinician, since these can only be purchased in bulk. The tissue will float off the template if the sponge is not used. Nail fragments may be placed in the same container outside of the tissue cassette.

## 9. Tips for Laboratory Processing of Nail Specimen

- Prior to sectioning, an initial assessment of the specimen by the pathologist is ideal. The clinical differential should guide grossing and sectioning.
- The entire cassette with the specimen on the template and the sponge should be fixed for a minimum of 8 h in formalin, after which it can be sent through overnight processing prior to sectioning, thereby allowing the specimen to become firmer to allow more precise sectioning.
- Embed small matrix/bed tissue segments in separate blocks if the specimen is fragmented.
- Cut initial 5 μm H&E level sections (three slides) with unstained charged slides for possible immunostains or other special stains. If you believe that the specimen may contain hard plate, then 4 μm should be cut instead. Valuable tissue can be lost when a block is refaced.
- Perform anticipated immunostains and other special stains with the initial sections. If they are not needed for the case, they can be discarded.

## 10. Submission of a Specimen of a Pigmented Band

The most common diagnosis for a pigmented band in the nail unit is “Benign activation of junctional melanocytes,” also known as “Melanotic macule” or “Benign lentigo” of the nail unit. The findings in this specific benign lesion are often focal and subtle. If the tissue is not processed carefully, the source of the clinical pigmentation may not be found. The laboratory should perform the following initial H&E and special stains when a pigmented band has been sampled.

## 11. Protocol for Initial Laboratory Work-Up of a Pigmented Band

- Level H&E sections (three slides)
- SOX-10 or MelanA (Mart-1) and PRAME immunostains to characterize melanocyte density and character
- Fontana–Masson stain to identify melanin; however, it must be diluted
- PAS stain to identify pigmented fungus as the cause of the pigmented band
- A few unstained sections on charged slides for possible additional H&E and special stains

**Figure 8:**
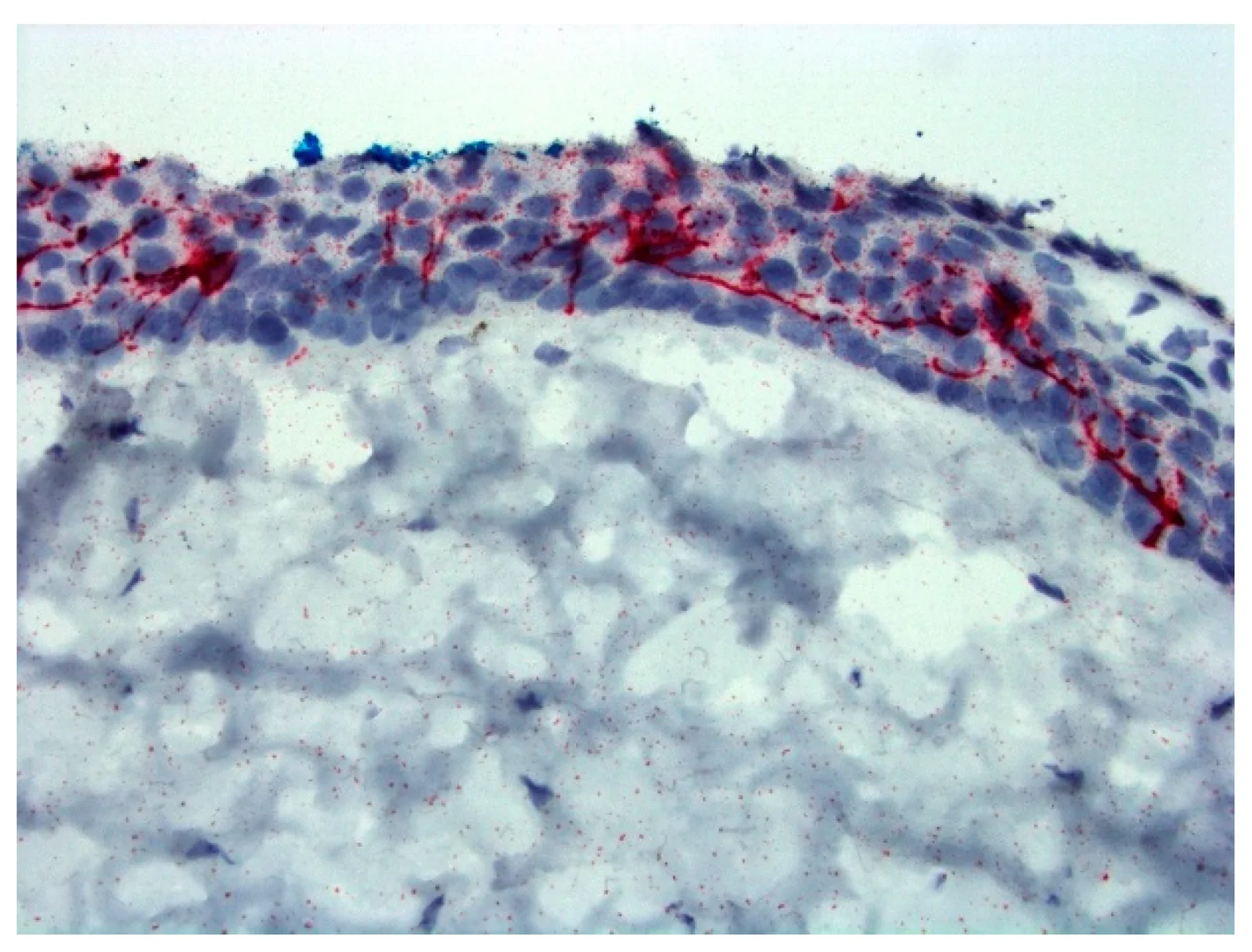
A MelanA/Mart-1 immunostain of a pigmented band matrix/bed specimen (400×).

Immunostains are quite helpful in assessing the melanocyte density in the epithelium, a feature which may not be evident on H&E sections. The use of MelanA/Mart-1, SOX-10 and PRAME immunostains requires correlation with the H&E histopathology, since they have recognized false-negative and false-positive rates (Figure 8). The Fontana–Masson stain will identify melanin within the nail plate; however, the stain must be diluted (hand stained) for use in the plate compared to other skin specimens.

It is optimal to have the nail plate for analysis since nail unit epithelium may remain adhered to the plate. However, if the clinician is adept at reflecting the nail plate, this is generally not the case. In addition, even though the plate will not re-adhere to the nail matrix/bed, clinicians often prefer to use the nail plate as a post-surgical protective barrier. If the epithelium remains attached to the nail plate and the plate is not submitted, there may be an inadequate sample. As mentioned in the section above, trauma-associated blood in the nail plate is the most common cause of a pigmented lesion.

## 12. Conclusions

By submitting and processing nail unit specimens using the specific protocols described above, significantly better H&E and special stain sections of both plate and matrix/bed are produced. With better processing, the pathologist can more accurately diagnose the variety of tumors, dermatitides and infections of the nail unit.

## Data Availability

The original contributions presented in this study are included in the article. Further inquiries can be directed to the corresponding author.
